# Non-Alcoholic Steatohepatitis (NASH): Risk Factors in Morbidly Obese Patients

**DOI:** 10.3390/ijms161025552

**Published:** 2015-10-23

**Authors:** Alexandre Losekann, Antonio C. Weston, Angelo A. de Mattos, Cristiane V. Tovo, Luis A. de Carli, Marilia B. Espindola, Sergio R. Pioner, Gabriela P. Coral

**Affiliations:** 1 Post-Graduation Program, Hepatology at Universidade Federal de Ciências da Saúde de Porto Alegre (UFCSPA), Porto Alegre 90.050-170, Brasil; E-Mails: alosekann@gmail.com (A.L.); angeloamattos@gmail.com (A.A.M.); cris.tovo@terra.com.br (C.V.T.); 2 Centro de Tratamento da Obesidade (CTO), Hospital Santa Casa de Misericórdia de Porto Alegre, Porto Alegre 92.010-300, Brasil; E-Mails: drweston@terra.com.br (A.C.W.); luizdecarli@plugin.com.br (L.A.C.); mariliae@brturbo.com.br (M.B.E.); srpioner@terra.com.br (S.R.P.)

**Keywords:** NAFLD, NASH, morbidly obese, liver fibrosis

## Abstract

The aim was to investigate the prevalence of non-alcoholic steatohepatitis (NASH) and risk factors for hepatic fibrosis in morbidly obese patients submitted to bariatric surgery. This retrospective study recruited all patients submitted to bariatric surgery from January 2007 to December 2012 at a reference attendance center of Southern Brazil. Clinical and biochemical data were studied as a function of the histological findings of liver biopsies done during the surgery. Steatosis was present in 226 (90.4%) and NASH in 176 (70.4%) cases. The diagnosis of cirrhosis was established in four cases (1.6%) and fibrosis in 108 (43.2%). Risk factors associated with NASH at multivariate analysis were alanine aminotransferase (ALT) >1.5 times the upper limit of normal (ULN); glucose ≥ 126 mg/dL and triglycerides ≥ 150 mg/dL. All patients with ALT ≥1.5 times the ULN had NASH. When the presence of fibrosis was analyzed, ALT > 1.5 times the ULN and triglycerides ≥ 150 mg/dL were risk factors, furthermore, there was an increase of 1% in the prevalence of fibrosis for each year of age increase. Not only steatosis, but NASH is a frequent finding in MO patients. In the present study, ALT ≥ 1.5 times the ULN identifies all patients with NASH, this finding needs to be further validated in other studies. Moreover, the presence of fibrosis was associated with ALT, triglycerides and age, identifying a subset of patients with more severe disease.

## 1. Introduction

Nonalcoholic fatty liver disease (NAFLD) embraces a wide range of manifestations that includes simple steatosis (SS), non-alcoholic steatohepatitis (NASH), cirrhosis and hepatocellular carcinoma [1,2]. The real prevalence of NASH is not known, as the disease is usually asymptomatic and that the definitive diagnosis is possible only by the histopathological assessment [3,4]. In a study conducted in a tertiary public hospital in south Brazil, the prevalence of NASH was 3.18% in obese patients without diabetes mellitus (DM) [5].

Morbidly obese (MO) patients, defined as body mass index (BMI) ≥35 and experiencing obesity-related health conditions or ≥40 kg/m^2^, are a subgroup with higher risk of NAFLD. In these patients, the prevalence of NAFLD is estimated from 84% to 96% and of NASH from 25% to 55%. In those with NASH, there is bridging fibrosis or cirrhosis at a rate of 12% and 2% respectively [4,6].

This study aimed to estimate the prevalence of NASH and the risk factors for fibrosis in MO patients submitted to bariatric surgery (BS).

## 2. Results

A total of 250 patients were evaluated; 200 (80%) were women, with an average age of 36.8 ± 10.2 years. The average BMI was 43.6 ± 5.2 kg/m^2^. Type 2 diabetes was identified in 12.8% and arterial hypertension in 41.3%.

Simple steatosis was present in 226 (90.4%) patients and were classified as mild in 76 (30.4%); moderate in 71 (28.4%) and severe in 79 (31.6%). NASH was diagnosed in 176 (70.4%) cases, being mild degree in 120 (48.4%) cases; moderate in 50 (20%) cases, and severe in 6 (2.4%) cases. Fibrosis was reported in 108 (43.2%) biopsies, 95 (38%) of them were mild; 2 (0.8%) moderate; and 7 severe (2.8%). Cirrhosis was diagnosed in 4 (1.6%) cases.

The risk factors related to NASH in bivariate analysis (Table 1) were: Mean value of AST, mean value of ALT, ALT ≥ 1.5 times the ULN, mean value of TG, TG ≥ 150 mg/dL and mean value of glucose. All patients with ALT ≥1.5 times the ULN had NASH. After the adjustment by the multivariate model, the following variables remain associated with NASH (Table 2): ALT > 1.5 times the ULN; glucose ≥ 126 mg/dL and TG ≥ 150 mg/dL.

Some risk factors associated to fibrosis by bivariate analysis (Table 3) were the same as those associated with NASH: Mean value of AST, mean value of ALT, ALT > 1.5 times the ULN, mean value of TG, TG ≥ 150 mg/dL and mean value of glucose. In addition, glucose ≥ 126 mg/dL and age were also associated with fibrosis. The mean age of patients with fibrosis was 40.0 ± 11.4 and without fibrosis, 34.8 ± 9.3 (*p* = 0.001). After the adjustment by the multivariate model (Table 2), the following variables remain associated with fibrosis: ALT > 1.5 times the ULN, TG ≥ 150 mg/dL and age: For a year of age increase, there is an increase of 1% in the prevalence of fibrosis (PR = 1.01; 95% CI = 1.00–1.02; *p* = 0.006).

**Table 1:** Bivariate analysis according to the presence of non-alcoholic steatohepatitis (NASH).

Variable *	Total Sample	With NASH	Without NASH	*p*
Age (years)	37.2 ± 10.6 (*n* = 183)	37.6 ± 11.0 (*n* = 141)	35.5 ± 9.0 (*n* = 42)	0.208
Female	153 (80.1) (*n* = 191)	113 (79) (*n* = 143)	40 (83.3) (*n* = 48)	0.661
BMI (kg/m^2^)	43.7 ± 5.2 (*n* = 191)	43.5 ± 5.0 (*n* = 143)	44.1 ± 5.7 (*n* = 48)	0.535
Ferritin (µ/L)	119 (67–208) (*n* = 169)	123 (75–239) (*n* = 128)	97 (58.5–173) (*n* = 41)	0.120
Iron (µ/L)	76.4 ± 25.2 (*n* = 163)	75.8 ± 24.1 (*n* = 125)	78.4 ± 29.1 (*n* = 38)	0.587
** AST (U/L)	24 (19–31) (*n* = 183)	25 (20–34) (*n* = 139)	21.5 (16.3–26.8) (*n* = 44)	0.007
** ALT (U/L)	29 (21–47.8) (*n* = 183)	32 (23–51) (*n* = 139)	25 (17–29.5) (*n* = 44)	<0.001
ALT > 1.5 × U/L	28 (15.2) (*n* = 183)	28 (20.1) (*n* = 139)	0 (0.0) (*n* = 44)	0.002
Glucose (mg/dL)	103.7 ± 34.3 (*n* = 188)	106.7 ± 37.7 (*n* = 142)	94.5 ± 17.9 (*n* = 46)	0.036
Glucose ≥ 126 mg/dL	24 (12.8) (*n* = 188)	22 (15.5) (*n* = 142)	2 (4.3) (*n* = 46)	0.086
Platelets (10³/mm^3^)	278.5 ± 68.6 (*n* = 172)	283.3 ± 64.8 (*n* = 131)	269 ± 68.8 (*n* = 41)	0.233
Total cholesterol (mg/dL)	193 ± 42 (*n* = 186)	196.6 ± 42.8 (*n* = 138)	182.9 ± 38.3 (*n* = 48)	0.052
LDL-C (mg/dL)	116 ± 41 (*n* = 186)	117.4 ± 41.1 (*n* = 138)	112 ± 41.1 (*n* = 48)	0.438
HDL-C (mg/dL)	48.9 ± 13.7 (*n* = 186)	48.4 ± 13.5 (*n* = 138)	50.2 ± 14.3 (*n* = 48)	0.427
TG (mg/dL)	122 (91–193) (*n* = 186)	134 (96–198) (*n* = 138)	105 (72–135) (*n* = 48)	0.004
TG ≥ 150 mg/dL	68 (36.3) (*n* = 186)	58 (42.0) (*n* = 138)	9 (18.8) (*n* = 48)	0.007

* Variables described by mean ± standard deviation, median (percentiles 25–75) or *n* (%); ** Normal values for ALT: 14–42 U/L and for AST: 10–42 U/L; *n* = number of cases; NASH = nonalcoholic steatohepatitis; BMI = body mass index; AST = aspartate aminotransferase; ALT = alanine aminotransferase; LDL-C = low density lipoprotein; HDL-C = high density lipoprotein.

**Table 2:** Multivariate analysis according to the presence of NASH and fibrosis.

Variables	NASH		Fb	
	PR (95% CI)	*p*	PR (95% CI)	*p*
ALT > 1.5 ULN	1.31 (1.22–1.41)	<0.001	1.22 (1.00–1.48)	0.048
Glucose ≥ 126 mg/dL	1.16 (1.02–1.32)	0.022	1.22 (0.99–1.50)	0.058
TGs ≥ 150 mg/dL	1.15 (1.01–1.30)	0.035	1.24 (1.07–1.45)	0.005
Age	*	*	1.01 (1.00–1.02)	0.006

* did not present a *p* value <0.20 in the bivariate analysis.

**Table 3:** Bivariate analysis according to the presence of fibrosis.

Variable *	With Fb	Without Fb	*p*
Age (years)	40.0 ± 11.4 (*n* = 83)	34.8 ± 9.3 (*n* = 100)	0.001
Female	67 (79.8) (*n* = 84)	86 (80.4) (*n* = 107)	1.000
BMI (kg/m^2^)	43.4 ± 5.4 (*n* = 84)	43.9 ± 5.0 (*n* = 107)	0.479
Ferritin (µ/L)	127 (81–293) (*n* = 73)	109 (56–97) (*n* = 96)	0.080
Iron (µ/L)	75.8 ± 22.5 (*n* = 73)	76.9 ± 27.4 (*n* = 90)	0.790
** AST (U/L)	25 (19–43) (*n* = 83)	24 (18–28) (*n* = 100)	0.040
** ALT (U/L)	30 (24–54) (*n* = 83)	26 (19–39) (*n* = 100)	0.008
** ALT > 1.5 × U/L	19 (22.9) (*n* = 83)	9 (8.9) (*n* = 100)	0.015
Glycemia (mg/dL)	110.9 ± 40 (*n* = 83)	98.0 ± 27.9 (*n* = 105)	0.014
Glycemia ≥ 126 mg/dL	17 (20.5) (*n* = 83)	7 (6.7) (*n* = 105)	0.009
Platelets (10³/mm^3^)	273.6 ± 59.3 (*n* = 77)	285 ± 70.6 (*n* = 95)	0.261
Total cholesterol (mg/dL)	198.9 ± 42.3 (*n* = 80)	188.7 ± 41.4 (*n* = 106)	0.102
LDL-C (mg/dL)	116.3 ± 38.4 (*n* = 80)	115.8 ± 43.1 (*n* = 106)	0.934
HDL-C (mg/dL)	49.2 ± 14.2 (*n* = 80)	48.6 ± 13.3 (*n* = 106)	0.776
TG (mg/dL)	148.5 (100–199) (*n* = 80)	112.5 (83.8–158) (*n* = 106)	0.005
TG ≥ 150 mg/dL	40 (50) (*n* = 80)	27 (25.5) (*n* = 106)	0.001

* Variables described by mean ± standard deviation, median (percentiles 25–75) or n (%); ** Normal values for ALT: 14–42 U/L and for AST: 10–42 U/L; *n* = number of cases; Fb = fibrosis; BMI = body mass index; AST = aspartate aminotransferase; ALT = alanine aminotransferase; LDL-C = low density lipoprotein; HDL-C = high density lipoprotein; TG = triglycerides.

## 3. Discussion

More recently, BS has become an accepted therapeutic option for MO patients and has been associated with histological improvement of NAFLD [7,8,9,10]. When liver biopsies performed before and after the weight loss caused by the surgery were compared, it was shown that this treatment determines an improvement or stabilization of SS, NASH and fibrosis [9,10]. However, in cirrhosis, the likelihood of regression is reduced and there is an increase in morbidity and mortality after BS [8,9,10,11,12].

In the present study, NAFLD was present in 90.4% of the MO patients submitted to BS. This result is consistent with the literature that reports a prevalence varying between 84% and 96% of NAFLD [4,13]. In the same way, the degree of steatosis was uniformly distributed in 30.4%, 28.4% and 31.6%, as mild, moderate and severe degree respectively, and NASH was found in approximately 70%, with a moderate correlation with the degree of steatosis. Other authors found a prevalence of NASH between 55% and 60%, but in these cases, the histopathological diagnostic criteria were not homogeneous, which makes the actual prevalence of NASH difficult to be established [3,11].

Bedossa *et al*. [14] proposed recently a score and algorithm for the histopathological definition of NASH in patients with MO. Patients should be classified as having NASH only if they have unequivocal hepatocyte ballooning. According to these criteria, a prevalence of NASH in 34% in patients with MO was found, which is lower than the observed in other studies [3,11], including ours. A possible explanation for this finding is that Bedossa *et al*. used more specific criteria for the diagnosis of NASH. In the present study, fibrosis was present in 48.3% of patients; out of these, 38% were mild and only 4.4% were considered severe. Although cirrhosis is not a contraindication for BS, there is a risk of hepatic decompensation with rapid weight loss [15].

New noninvasive clinical and biochemical markers of fibrosis in NASH have been evaluated [3]. Age, obesity, hypertension, DM, the levels of bilirubin and the ALT/AST ratio greater than 1 has been associated with the presence of NASH or fibrosis [3,13,16,17,18]. Contrary to other studies [19,20], the present results did not show a positive correlation of BMI with the degree of steatosis, NASH and fibrosis. BMI does not always properly reflect the degree of visceral adiposity, significantly more involved in the physiopathology of NAFLD. It is possible that there is a closer correlation between the liver damage and the measure of abdominal circumference; however, this data was not evaluated in the present study.

The results of the present study demonstrated that all patients whose ALT values were greater than 1.5 times the ULN (15% of the sample) presented NASH, and ALT was also strongly associated with fibrosis. This data can represent a cutoff and has not yet been reported in the literature for this subgroup of patients.

This study showed an association among serum levels of TG and glucose with NASH. These findings were already described in former studies concerning the risk factors of NASH [13,18,21]. In addition to high levels of TG, we found that the presence of fibrosis was also correlated with age; this association has been described before [20,22]. Furthermore, an increase in age raises the prevalence of fibrosis linearly.

Although several non-invasive markers for prediction of advanced fibrosis are available (aspartate aminotransferase-to-platelet ratio index - APRI; NAFLD fibrosis score; body mass index, ASL/ALT ratio and diabetes mellitus - BARD; FIB-4) [16,23,24,25], the present study suggests that patients with MO and more advanced age, high levels of ALT and TG should best be submitted to a full diagnostic evaluation such as liver biopsy to better assessment of hepatic damage.

In conclusion, this study showed a high prevalence of NASH in patients with MO and identifies a subset of patients with a higher risk of more advanced disease.

## 4. Experimental Section

This is a retrospective cohort study, where MO patients were submitted to BS from 2007 to 2012 at the Obesity Treatment Center of a tertiary reference center (Santa Casa de Porto Alegre, SCPA) in southern Brazil. Age, gender, the presence of comorbidities (diabetes, arterial hypertension) and body mass index (BMI) were evaluated. The dosage of ferritin, aspartate (AST) and alanine (ALT) aminotransferases, fasting glucose, platelets, total cholesterol, triglycerides (TG), high (HDL-C) and low (LDL-C) density lipoproteins was done up to 90 days before procedure. These variables were compared with the histological results of liver biopsies obtained in the trans-operative period.

Patients aged less than 18 years, those who presented serological markers for viral hepatitis, as well as patients with other causes of chronic liver disease and history of alcohol intake >20 g/day were excluded.

Liver biopsies were routinely stained with Hematoxylin-Eosin, Perls and Masson’s trichrome and evaluated by the same liver pathologist who was blinded to the clinical data.

Simple steatosis (SS) was considered to be present over 5% of the sample and scored as suggested by Brunt: Mild steatosis was defined when present in 5% to 33%; moderate steatosis when present in 33% to 66%, and severe steatosis when greater than 66% [26]. To diagnose NASH, steatosis associated with hepatocyte ballooning and/or inflammatory infiltrate were the main findings, and was classified using NAFLD Activity Score (NAS) as mild (A1), moderate (A2) and severe (A3), according to classification described by the Pathology Committee of the NASH Clinical Research Network. The degree of fibrosis (Fb) was classified as stage A1, when sinusoidal/discrete cellular Fb was present; degree 1B, when sinusoidal/dense and diffuse Fb was identified; and 1c for portal Fb. Stage 2 was considered when there was pericellular/perisinusoidal associated with periportal Fb, and stage 3 in the presence of the anterior changes associated to bridging Fb. Finally, stage 4 corresponds to cirrhosis [27]. In the statistical analysis, the degree of Fb was classified as mild (stages 1A, 1B, 1C); moderate (stage 2); severe (stage 3) or cirrhosis (stage 4).

The data were analyzed using the SPSS (Statistical Package for the Social Sciences) Inc., Chicago, IL, USA, version 18.0. The sample size supports a minimum difference between groups of 20%, power of 85% and a significance level of 5%. To control confounding factors and analyze the variables independently associated with NASH and fibrosis, the Poisson regression analysis was applied. To evaluate the association, the prevalence ratio (PR) was used, with the 95% confidence interval (CI) to estimate the risk in the population. To control the multicollinearity, two regression models were made, one of them inserting the glycemia and the other the TG. The criteria for entering the variable in the multivariate model was that it should have a value of *p* < 0.20 in the bivariate analysis. To evaluate the association between the categorical variables, the Pearson chi-square test was applied, and for the continuous or ordinal variables, the Spearman (r_s_) correlation test was used. *p* values of <0.05 were considered significant. This study was approved by the Institutional review board of SCPA. For this type of study formal consent was not required.

## Abbreviations

ALT: alanine aminotransferase; AST: aspartate aminotransferase; APRI: aspartate aminotransferase-to-platelet ratio index; BARD: body mass index, ASL/ALT ratio and diabetes mellitus; BMI: body mass index; BS: bariatric surgery; CI: confidence interval; DM: diabetes mellitus; Fb: fibrosis; HDL-C: high density lipoproteins; LDL-C: low density lipoproteins; MO: morbidly obese; NAFLD: Nonalcoholic fatty liver disease; NAS: NAFLD Activity Score; NASH: non-alcoholic steatohepatitis; PR: prevalence ratio; r_s_:Spearman correlation test; SCPA: Santa Casa de Porto Alegre; SPSS: Statistical Package for the Social Sciences; SS: simple steatosis; TG: triglycerides; ULN: upper limit of normal.
